# Supplementary material for: High Dose of Dietary Nicotinamide Riboside Induces Glucose Intolerance and White Adipose Tissue Dysfunction in Mice Fed a Mildly Obesogenic Diet
Source: Nutrients. 2019 Oct 13;11(10):2439. doi: 10.3390/nu11102439 (PMC6835358; doi:10.3390/nu11102439)
Supplement: Supplementary file 1 [file nutrients-11-02439-s001.pdf]

Supplemental Tables

Table S1. Composition of diets

Ingredients (g·kg-1 diet)	Adaptation	Ctrl NR	High NR
Casein	100.0	100.0	100.0
Wheat starch	233.1	233.1	233.1
Gelatin (hydrolysed)	100.0	100.0	100.0
Maltodextrin	100.0	100.0	100.0
Sugar	100.0	100.0	100.0
Dextrose	50.0	50.0	50.0
Arbocel B800	50.0	50.0	50.0
Linseed oil	4.0	4.0	4.0
Palm oil	206.0	206.0	206.0
Mineral mixture AIN-93	35.0	35.0	35.0
Vitamin mixture AIN-93[a]	10.0	10.0	10.0
L-Cystine	3.25	3.25	3.25
L-Phenylalanine	4.32	4.32	4.32
Choline chloride 50%	1.55	1.55	1.55
Nicotinamideriboside (mg·kg-1 diet)	30.0	30.0	9000.0
Calculated amount of L-tryptophan (%)	1.15	1.15	1.15
Calculated energy (kcal·kg-1)	4653	4653	4653
Carbohydrate (% of total energy)	41	41	41
Fat (% of total energy)	40	40	40
Protein (% of total energy)	19	19	19

a Vitamin B3 withdrawn

Table S2. Sequences of primers for qRT-PCR [a]

Gene symbol	Primer forward 5’-3’	Primer reverse 5’-3’
B2m [b]	CCCCACTGAGACTGATACATACGC	AGAAACTGGATTTGTAATTAAGCAGGTTC
C3	AAAGATTTCACACACCGAAGAAGACTG	GAGCATCCCATCGTCCTTCTCTG
Casp1	CCATGGCTGACAAGATCCTGAG	CATAGGTCCCGTGCCTTGTC
Cfd (*Adipsin*)	TCACCATTAACATGATGTGTGCAGAG	GGATGACACTCGGGTATAGACGC
Grb14	CGGTCCCAGCCATGGTTTCAC	GTTACTCTGACTATCCCGTACC
Insr	CATCATGTGGTCCGCCTTCT	CCGGTGCACAAACTTCTTGG
Irs1	TTAGGCAGCAATGAGGGCAA	TCTTCATTCTGCTGTGATGTCCA
Irs2	GCACCTATGCAAGCATCGAC	GCGCTTCACTCTTTCACGAC
Itgad (Cd11d)	TTAGGCAGCAATGAGGGCAA	TCTTCATTCTGCTGTGATGTCCA
Itgax (Cd11c)	GTTTGAGTGTCAGGAGCAGGT	GAGGTCACCTAGTTGGGTCTTG
Pck1	GTTTGTAGGAGCAGCCATGAGATC	CCAGAGGAACTTGCCATCTTTGTC
Pparg (Pparγ)	GAAGTTCAATGCACTGGAATTAGATGAC	TTGTCTTGGATGTCCTCGATGGG
Rps15 [b]	CGGAGATGGTGGGTAGCATGG	ACGGGTTTGTAGGTGATGGAGAAC
S100a8	ACTTCGAGGAGTTCCTTGCG	TGCTACTCCTTGTGGCTGTC
Saa1	AGACACCAGGATGAAGCTACT	AAGGCCTCTCTTCCATCACT
Saa3	AAAGAAGCTGGTCAAGGGTC	TGTCCCGTGAACTTCTGAAC
Scd1	TCATGGTCCTGCTGCACTTGG	CTGTGGCTCCAGAGGCGATG
Slc2a4 (Glut4)	CCATTCCCTGGTTCATTGTG	GTTTTGCCCCTCAGTCATTC

[a] All the primers were used with the optimal annealing temperature at 60°C.

[b] Reference mRNAs.

Table S3. Effects of High NR on circulating indicators for WAT function [a]

Indicators	Ctrl NR [b]		High NR		p value [c]
	mean	SEM	mean	SEM	
Blood glucose (mmol/L)	5.9	0.2	5.5	0.2	0.173
TG (mg/dL)	131.6	7.0	131.3	4.0	0.976
NEFA (mmol/L)	1.1	0.1	0.9	0.1	0.342
Total cholesterol (mg/dL)	134.8	9.9	165.2	9.1	0.035
HDL cholesterol (mg/dL)	75.4	8.7	77.4	5.1	0.841
LDL cholesterol (mg/dL)	43.9	6.2	66.8	9.1	0.055
Leptin (mg/mL)	1.3	0.2	1.7	0.2	0.102
Adiponectin (mg/mL)	1.1	0.3	0.8	0.1	0.356

[a] Samples were collected from animals which were in a postprandial state.

[b] Ctrl NR, 30NR; High NR, 9000NR. NR in mg per kg diet.

[c] Data are analysed using Student's *t*-test (n=10-12 mice per treatment).

Supplementary Figures

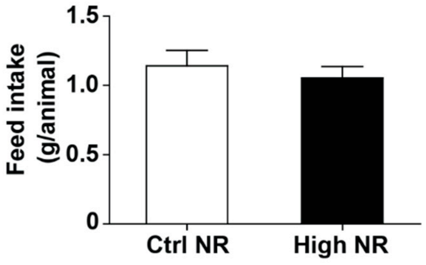

Figure S1. No difference in feed intake before dissection. Individual feed intake before the dissection was measured in week 18. Ctrl NR white bar, High NR black bar. Data are analyzed using Student's *t*-test and shown as mean ± SEM (n=11-12 mice per treatment).

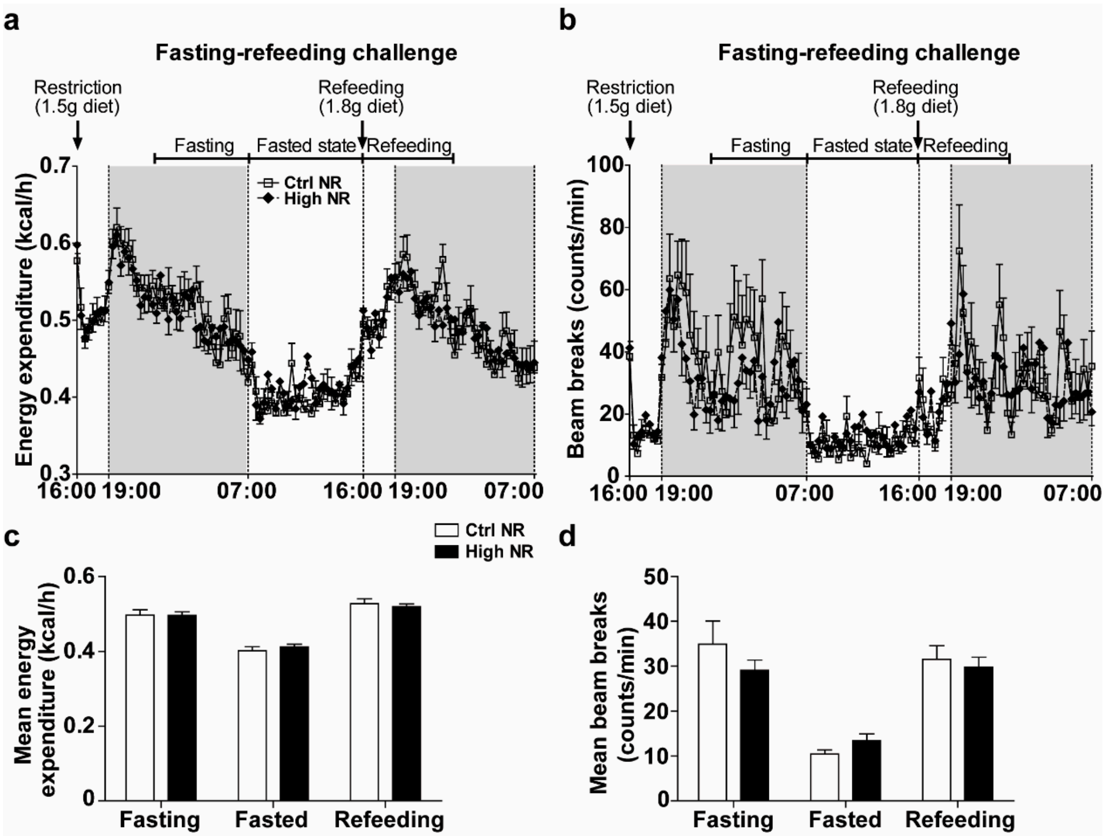

Figure S2. Energy expenditure and physical activity do not differ between Ctrl NR and High NR. Energy expenditure (**a**) and real-time physical activity (indicated as beam breaks per min, (**b**) during the fast-refeeding challenge were measured using indirect calorimetry in week 14: restriction with 1.5 gram of diet at 16:00h, fasting (from 22:00h to 07:00h), fasted (from 07:00h to 16:00h), refeeding with 1.8 gram of diet at the next 16:00h, refeeding (from 16:00h to 23:00h) periods. Shaded areas indicate the dark, active periods. Mean energy expenditure (**c**) and beam breaks (**d**) were analyzed for each period. Ctrl NR (30NR) open square with solid line or white bar, High NR (9000NR) closed diamond with black dashed line or black bar. NR in mg per kg diet. Data are analyzed using two-way repeated measures ANOVA followed by Bonferroni post-hoc analysis and shown as mean ± SEM (n=11-12 mice per treatment).

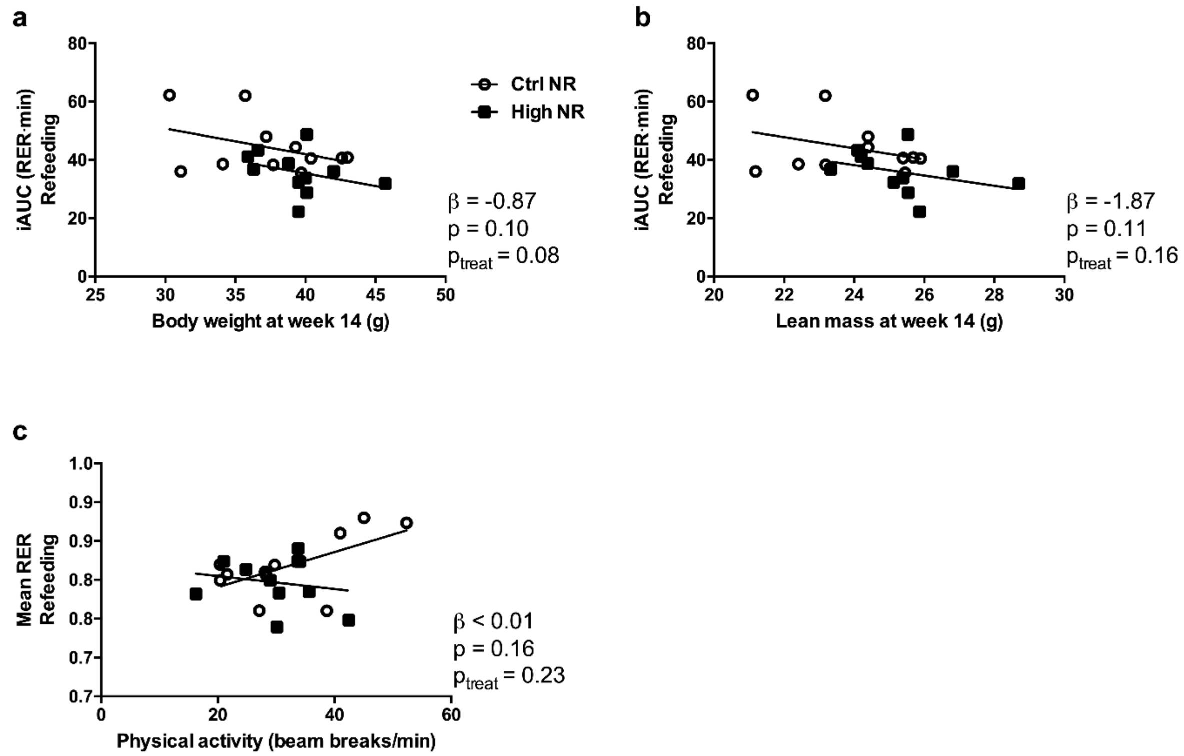

Figure S3. Difference in metabolic flexibility between two treatments was not related to their body weight, lean mass or activity during fasting-refeeding in indirect calorimetry test. Regression analysis for individual animals was performed between iAUC of RER and body weight (**a**) or lean mass (**b**), or between mean RER and activity (**c**) (n=11 mice per treatment).

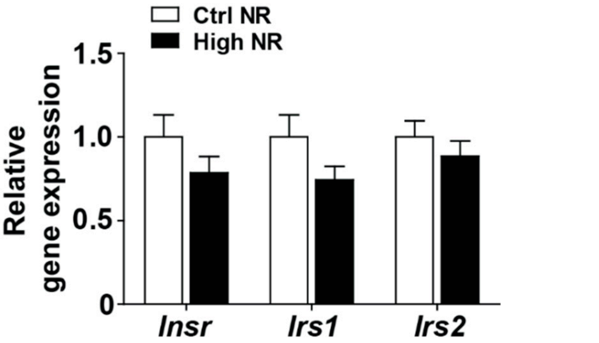

Figure S4. High NR does not alter insulin receptor genes in eWAT. Relative gene expression (normalized to the reference genes) of insulin receptor genes. Ctrl NR white bar, High NR black bar. Data are analyzed using Student's *t*-test and shown as mean ± SEM (n=11 mice per treatment).

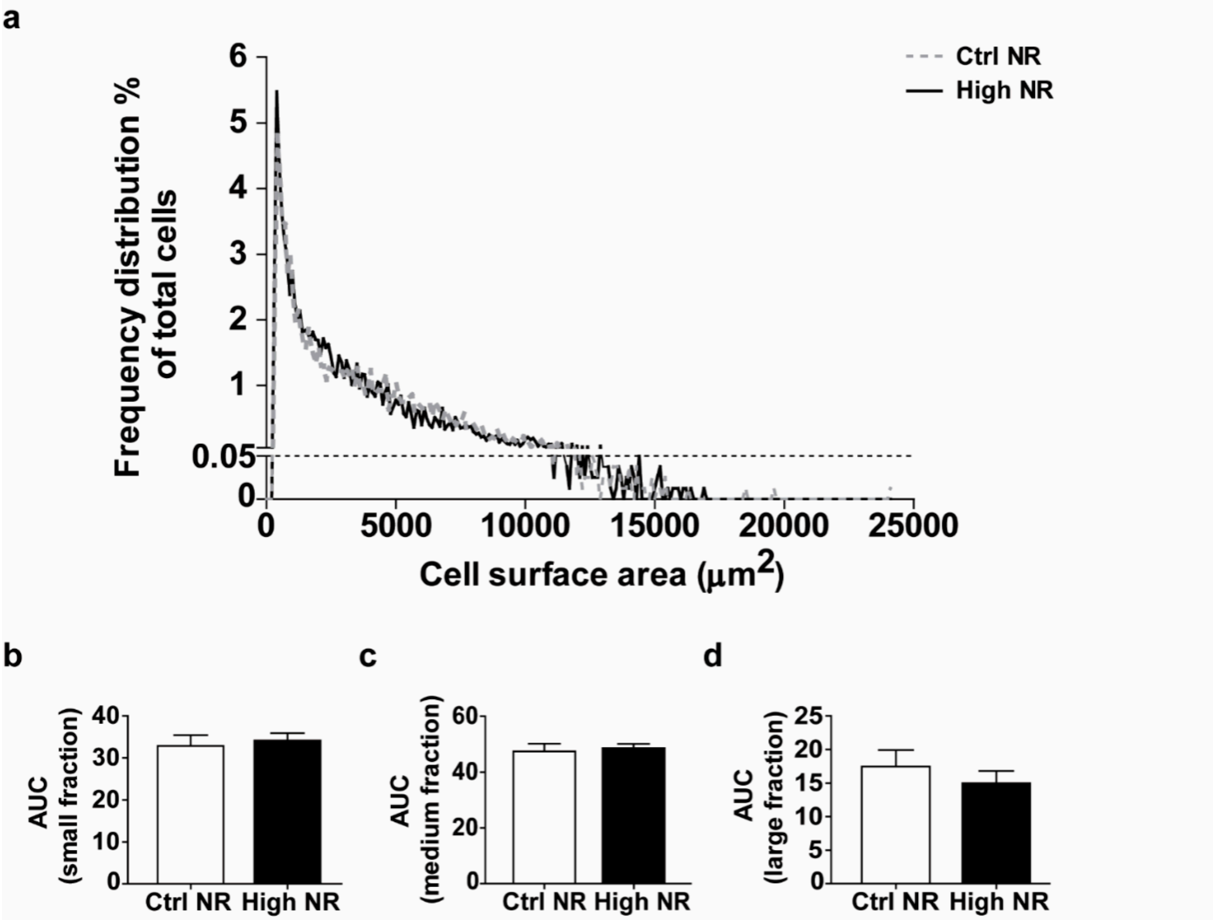

Figure S5. Adipocyte size frequency distribution is comparable between Ctrl NR and High NR. Frequency distribution (**a**) and area under the curve (AUC) of small (100-1500µm^2, **b**), medium (<1500-6000µm^2, **c**) and large (>6000µm^2, **d**) adipocyte fractions. Ctrl NR grey solid line or white bar, High NR black dashed line or black bar. Data are analyzed using Student's *t*-test and shown as mean ± SEM (n=8 mice per treatment).

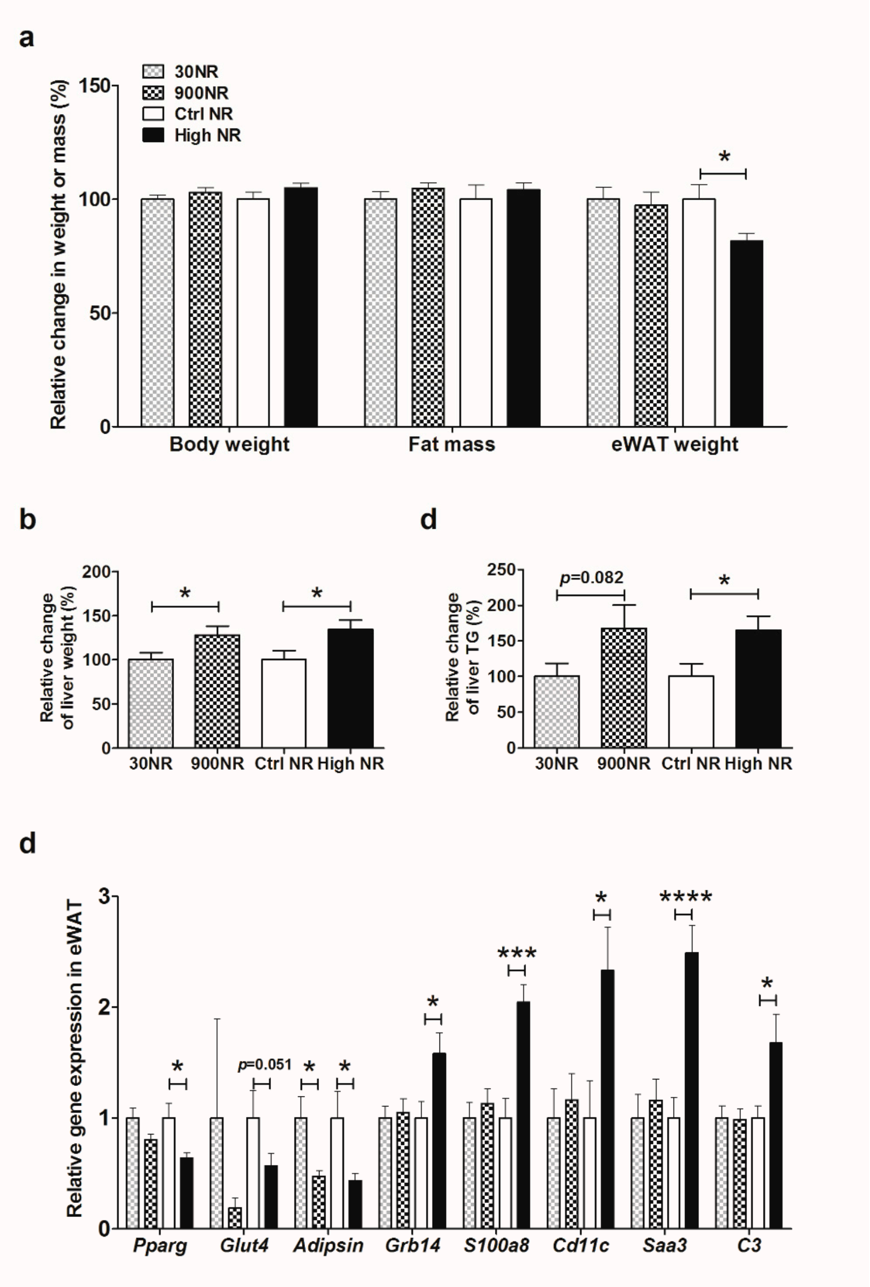

Figure S6. Dose-response effects of dietary NR at high doses. Body weight and fat mass of animals were measured at the end of the study and after necropsy liver and eWAT were quickly dissected and weighed. Changes in body weight, fat mass and eWAT weight of animals treated with high doses of dietary NR relative to their control cohorts were calculated and shown as percentage of change with control set at 100% (**a**). Relative change of liver weight (**b**) and liver triglycerides (TG) content (**c**). Relative gene expression in eWAT (normalized to the reference genes) of genes involving adipogenesis, insulin signalling and pro-inflammatory response (**d**). 30NR (equivalent to Ctrl NR in the present study) grey dotted bar, 900NR black dotted bar; Ctrl NR (= 30NR) white bar, High NR (= 9000NR) black bar. NR in mg per kg diet. Data are analyzed using Student's *t*-test and shown as mean ± SEM (n=11-12 mice per treatment). Data of body weight and fat mass, as well as relative gene expression of *Pparg* and *Glut4* for 30NR and 900NR have been published in *Shi W, et al., 2017, doi: 10.1002/mnfr.201600878,* while data of eWAT weight, liver weight, liver TG content as well as

relative gene expression of *Adipsin*, *Grb14*, *S100a8*, *Cd11c*, *Saa3* and *C3* for 30NR and 900NR were newly analyzed together with the same parameters of this study.
